# Influence of dietary *Salicornia europaea* L. extract supplementation on feed efficiency of Altay sheep by modifying their gastrointestinal bacteria communities

**DOI:** 10.3389/fmicb.2024.1377314

**Published:** 2024-04-15

**Authors:** Mahmoud Kamal, Wang Lele, Tang Shuzhen, Liang Jiandi, Qin Rongyan, Liu Yanfeng, Wang Wenqi, Chen Xiangyu, Yanfen Cheng

**Affiliations:** ^1^Feed Research Institute, Xinjiang Academy of Animal Sciences, Ürumqi, China; ^2^Laboratory of Gastrointestinal Microbiology, National Center for International Research on Animal Gut Nutrition, Nanjing Agricultural University, Nanjing, China; ^3^Animal Production Research Institute, Agricultural Research Center, Giza, Egypt; ^4^Key Laboratory of Xinjiang Feed Biotechnology, Ürumqi, China

**Keywords:** *Salicornia europaea*, growth performance, fermentation, microbiota, sheep

## Abstract

This experiment aimed to examine the impact of *Salicornia europaea* L. extract on sheep growth performance, rumen fermentation variables, nutrient apparent digestibility, and gastrointestinal microbial diversity. Forty-eight male Altay sheep, weighing 32.5 ± 2.8 kg and approximately 3.5 months old, were chosen. Four dietary treatments, each consisting of four replicates and three sheep per replicate, were distributed randomly to the sheep. The pelleted total mixed ration containing *Salicornia europaea* L. extract at 0.0, 0.2, 0.4, and 0.6% DM was freely available to the sheep in the four treatment groups. The 56-day experiment consisted of 45 days of measurements followed by 11 days of adaptation. The growth performance was not affected by nutrition *Salicornia europaea* L. extract (*p* ≤ 0.05), but the feed-to-gain ratio was reduced when the extract was given at 0.4% DM (*p* ≤ 0.05). Compared to the 0 and 0.2% treatments, the apparent digestibility of DM, OM, NDF, and ADF was substantially greater in the 0.4, and 0.6% treatments. Furthermore, compared to sheep in the 0 and 0.2% groups, sheep in the 0.6% group had a noticeably higher apparent digestibility of CP. As the amount of *Salicornia europaea* L. extract added to the rumen fluid rose, the molar ratio of acetic acid increased. In contrast, the molar ratio of propionic acid gradually decreased, and the total volatile fatty acid content gradually reduced. Thus, adding a suitable quantity of *Salicornia europaea* L. extract to the sheep ration is natural and secure, which may improve the environmental sustainability of small ruminant production systems.

## Introduction

Livestock products are experiencing a surge in demand due to income growth, global population increase, and rising urbanization, particularly in developing nations. According to Cheng et al. (2022) and Liu et al. (2023), 33 percent of the world’s protein and 17 percent of the world’s calories come from livestock. Predictions suggest that by 2050, the consumption of animal products could rise by 70% due to escalating urbanization, income levels, and global population growth (Yitbarek, 2019). Meeting this increased demand will necessitate the production of more animal feed. Some countries may face a shortage of forage for ruminants because of climate change, leading to water supply deficiencies and increased salinity in soil and groundwater (Singh et al., 2014; Khondoker et al., 2023).

Under these challenging circumstances, halophyte crops that are acclimated to high temperatures, saltiness in the soil and water, and other factors might provide adequate fodder (Abouheif et al., 2000; Abdellaoui et al., 2023). Because they can thrive in highly salinized conditions, halophytes hold promise for enhancing ecosystems and saline agricultural methods. It may preserve freshwater, produce valuable goods, recover biodiversity, and enhance soil quality. Halophytes have been successfully employed for restoring coastal habitats and wetlands, and they may offer important genes for biosaline agriculture (Cheeseman, 2015; Ventura et al., 2015). Through yields of up to 23.1 t DM/ha, the Salicornia species, which belong to the Amaranthaceous family, are succulent halophytic herbs with considerable economic potential as alternative crops. Water with a salt content as high as seawater can be used to irrigate it, and after the plant grows, the Na salts can be removed to aid in the restoration of saline soils (Chaudhary et al., 2018). Badri and Ludidi (2020) found important variation among halophyte species, which helped improve conventional fodder plants, provide animal feed, conserve fresh water in African nations, and understand the biological processes and genetic tolerances toward environmental constraints.

*Salicornia europaea* L. is a Mediterranean halophytic plant indigenous to East Asia. It can withstand salt stress thanks to an abundance of bioactive defense secondary metabolites. In Korea and Europe, *Salicornia europaea* is frequently eaten as a raw vegetable or as a fermented food. It is now available for purchase as an edible halophyte (Kang et al., 2015; Gunning, 2016). *Salicornia europaea* L. has been found to contain carbohydrates, proteins, minerals, oils, phenolic compounds, flavonoids, sterols, saponins, alkaloids, and tannins according to phytochemical studies (Patel, 2016). Furthermore, *Salicornia europaea* L. may be used therapeutically as an antibacterial, anti-oxidative, anti-inflammatory, anti-adaptogenic, anti-vascular neointima, anti-hyperlipidemic, and anti-diabetic agent, according to mounting data (Hwang et al., 2010). The crude protein (CP) levels of Salicornia species range widely, from 60 to 200 g/kg DM, while the ash ranges from 140 to 300 g/kg dry matter (DM) (El Shaer, 2010). Furthermore, it was reported that Salicornia has a low lignin content (19.6 g/kg, DM basis) (Bañuelos et al., 2018). Also, Akinshina (2014) examined seven distinct halophyte plants, finding that each had 140–490 g of ash/kg of dry matter. Salicornia had an ash content of 485.52 ± 10.11 g/kg of DM.

Because residual solvents may be harmful to the health of humans, organic materials extracted with water, ethanol, or their mixtures are being used more and more as food additives in a variety of foods and as preventative or therapeutic agents (Awad et al., 2021). The hot water method of extraction is thought to be the most practical and straightforward of all the extraction techniques (Jin et al., 2023). Even though there are a ton of research investigations on the properties of halophyte essential oil extracts made with toxic solvents (acetone, methanol, and chloroformic acid) and the chemical makeup of these oils (Joseph et al., 2013; Jallali et al., 2014), environmentally friendly extraction methods like UAE or SFE—which only require ethanol or water—are not being used. Cristina et al. (2018) used supercritical fluid extraction and ultrasound-assisted extraction to study extracts of sea fennel and marsh samphire from Mediterranean coast plants. The ideal temperature, ethanol concentration, was at 50°C and 300 bar pressures with 40% (v/v) ethanol concentration, and extraction time (20 min), and UAE and SFE extraction parameters were 50°C and 20 min, respectively. *Salicornia europaea* L. had the least number of antioxidants, whereas the extract from *Crithmum maritimum* L. had the highest amount.

According to Mahmoud et al. (2016), the feed performance of calf camels can be negatively impacted by using Salicornia biomass as a roughage component up to 25% in a diet that includes no more than 14.5% crude protein. This plant’s main component is NaCl, which contributes to its high ash content (Zhang et al., 2015). Elevated sodium levels in the diet lead to a rise in water consumption and the rumen’s flow of undigested digest, which improves protein digestion and decreases the fermentation of fiber (Basmaeil et al., 2003). Additionally, the negative effects of high ruminal fluid osmolality on microbial growth and lowered ruminal digestive juices retention time, the elevated salt levels in Salicornia forage raised water consumption, which in turn decreased digestion (Masters et al., 2007). According to Taghipour et al. (2021), adding *Salicornia bigelovii* forage up to 30% of it instead of alfalfa hay to a sheep’s maintenance diet raised blood’s total antioxidant capacity and reduced *in vitro* ruminal methane production without hurting the nutrient digestibility of the animal *in vivo*.

This study evaluated the impact of feeding *Salicornia europaea* L. extract on the health status, rumen fermentation parameters, nutrient digestibility, growth performance, and gastrointestinal microbial diversity of Altay sheep.

## Materials and methods

### Ethical approval

The protocols and methods used in the experiments were authorized by the Xinjiang Animal Welfare and Ethics Committee Academy of Animal Sciences, China (Approval No., 2021–276, 15 May 2021). All animal treatments and experiments were performed according to the recommendations of the guidelines for ethical review of animal welfare in the national standards of the People’s Republic of China.

### Animals and the administration

Forty-eight Altay male sheep with body weights of (32.5 ± 2.8 kg) at the age of 3.5 months were selected and purchased from Xinjiang Zhongxin Qi Agricultural and Animal Husbandry Professional Cooperatives Association. The basal diet (Table 1) consisted of a complete mixed diet with a concentrate-to-forage ratio of 65:35 designed to meet the Daily Nutrient Requirements of Meat Sheep (DB65/T 4244–2019) requirements for growing sheep.

**Table 1:** Composition and nutritional level of basal diet (DM basis) %.

Ingredients	Content %	Nutrient composition %	Content %
Corn	28.00	Metabolic energy(MJ/kg)	9.82
Soybean meal	27.00	Dry matter	93.97
Wheat bran	7.00	Organic matter	84.88
CaHPO_4_	0.50	Crude protein	16.80
Limestone	1.00	Ether extract	1.59
NaCl	1.00	Neutral detergent fiber	33.58
Premix^1^	0.50	Acid detergent fiber	14.54
Alfalfa hay	25.00	Calcium	0.42
Straw	5.00	Total phosphorus	0.12
Wheat hay	5.00		
Total	100.00		

^1^Provided the following (per kilogram of complete diets): VA 5000 IU, VD 600 IU, VE 16 IU, Cu (CuSO_4_·5H_2_O) 21.5 mg, Zn (ZnSO_4_·H_2_O) 90 mg, Co (CoCl_2_·6H_2_O) 1.1 mg, Mn (MnSO^4^·5H_2_O) 80 mg, Fe (FeSO^4^·7H_2_O) 12 mg, I (KI)1.2 mg.

Animal testing was conducted in the Xinjiang Zhongxin Qi Agricultural and Animal Husbandry Professional Cooperatives Association, and the animal house was thoroughly cleaned and disinfected before the purchase of test animals. Each experimental animal was raised in a single column (1.0 m × 1.2 m) and fed twice a day at 9:00 AM and 6:00 PM respectively, during which time the experimental animals were free to eat and drink.

The *Salicornia europaea* L. extract was provided by Henan Yixin Biotechnology Co., Ltd. The collected fresh stem and leaf samples of *Salicornia europaea* L. were dried, crushed, and mixed with a certain volume of distilled water. Then it was extracted in an ultrasonic-assisted extraction machine, and the crude extract was concentrated and spray-dried to obtain the *Salicornia europaea* L. extract.

### Trial design

Using the equivalent body weight principle, the sheep were split into four dietary treatments at random, with four replicates for each treatment and three sheep per replicate. The first group served as a control group and was fed only the basal diet. The other three experimental groups were fed the basal diet supplemented with *Salicornia europaea* L. extract at 0.2, 0.4, or 0.6% DM, respectively. The feeding experiment lasted 56 days.

### Measurements

#### Growth performance

Every 3–4 days, the amount of feed was changed to allow for five to 10 % residues. The weight gain of individual sheep was measured from the 1st and 56th day to the end of the experiment. The daily feed intake and leftover feed amount for each repeated group of sheep were recorded. The daily intake of dry matter and average daily gain (ADG) were calculated; the feed gain (F/G) ratio was calculated based on the ADG and daily dry matter intake.

### The measurement of apparent digestibility

Offered and refused feed were recorded daily; during the last week of the experiment, five sheep from each treatment with similar DMI and BW were selected for a 7-day digestibility test. The nutrient’s apparent digestibility was determined by the acid-insoluble ash method. The feces of each sheep were collected by the total manure collection method twice a day. After mixing, 10% of the feces were taken as mixed samples and stored in a zip locked bag. Ten mL of nitrogen fixation (10% diluted sulfuric acid) was added to every 100 g of fresh feces during 5 days of continuous feces collection. Every sheep’s feces sample was combined individually and frozen at −20°C.

ANKOM 200i fiber analyzer (ANKOM Technologies, Inc., Fairport, NY, United States) was used to analyze the contents of the DM, organic matter (OM), and CP by AOAC (2000). The neutral detergent fiber (NDF) and acid detergent fiber (ADF) were analyzed using the ANKOM filter bag technique. Nutrient digestibility = 100–100 x (% indicator in feed x % nutrient in feces)/(% indicator in feces x % nutrient in feed) was the model used for determining digestion (Huhtanen et al., 1994).

### Rumen fermentation indicators

Samples of rumen fluid were taken after the digestibility test was completed. Before the morning nutrition, five sheep from each group had their rumen fluid collected. Following the pooling and filtering of this fluid through four layers of cheesecloth, a 100 mL sample of rumen fluid was taken, then thoroughly combined, split into 10 mL centrifuge tubes, stored in liquid nitrogen, and finally stored at −80°C. A digital pH meter (Testo205 type) was utilized to determine the ruminal pH. The test consisted of a solution of phenol–hypochlorite and was utilized to ascertain the concentration of ammonia-N (NH_3_-N). Gas chromatography (Agilent 6,890 N Gas Chromatograph) fitted with a capillary column (19,091 N-213; Agilent) was used to measure the volatile fatty acids (VFA) in rumen.

### Ruminal and intestinal microbiota

Following the completion of the digestibility evaluation, rectal feces samples were collected. From each group, five sheep were chosen, and samples of the rectal feces were artificially collected. These samples were then frozen at −80°C in a cryogenic vial for additional examination. After collecting and nominating a portion of the rumen liquid through the layers of lumbar fabric, some of the samples were filtered via four layers of muslin cloth, collected in 50 mL falcon tubes labeled with the relevant information, and then subjected to bacterial genomic DNA extraction.

### Ruminal and intestinal microorganism DNA extraction

Following the kit’s instructions, samples were used to extract microbial genomic DNA (Tian Gen Biochemical Technology Co., Ltd.). A 1% agarose gel electrophoresis was used to assess the extraction quality, and a micro-ultraviolet spectrophotometer (Nanodrop 2000) was utilized to measure the purity and concentration of DNA. For future use, the extracted whole genomic DNA was kept at −20°C.

### PCR amplification and sequencing

The construction and sequencing of bacterial genomic libraries were completed using Shanghai Personal Biotechnology Co. Ltd., using PCR to amplify the standard bacterial 16S rRNA V3–V4 region. The bacterial universal primer sequences used were 338F (5’-ACTCCTACGGGAGGCAGCAG-3′) and 806R (5′ -GGACTACHVGGGTWTCTAAT-3′). The amplification system consisted of 25 μL of reaction buffer, GC buffer, dNTP, forward and reverse primers, DNA template, ddH2O, and Q5 DNA Polymerase. The PCR products underwent 2% agarose gel electrophoresis and were recovered using the Axygen gel recovery and a purification kit was used to recover the V3-V4 region amplification products.

The TruSeq Nano DNA LT Library Prep Kit from Illumina was used to create the Illumina sequencing library. The Agilent High Sensitivity DNA Kit was used to assess the library’s quality on the Agilent Bioanalyzer before sequencing. The Quant-IT Pico Green dsDNA Assay Kit was then used to quantify the library utilizing the Promega Quanti Fluor fluorescence quantitative system. Mi Seq Reagent Kit V3 was used to sequence the qualified library, requiring 600 cycles.

High-throughput sequencing data was preliminarily screened for quality, retested, and regrouped into libraries and samples. Quality control, denoising, ligation, and chimera removal were performed using the QIIME2 dada2 analysis process to obtain ASVs. The QIIME2 classified-sklearn algorithm and Naive Bayes classifier were used to annotate taxonomic levels of species, the indexes of species richness, diversity, and evenness (chao1, goods coverage, observed species, Shannon, Simpson), and calculate distance matrices. Using unsupervised and supervised methods, the PCoA analysis method was used to measure β diversity and species taxonomic composition in experimental groups. Attempts were made to identify marker species and measure differences in abundance composition among experimental groups.

### Statistical analysis

ANOVA was utilized to analyze growth performance, digestibility, metabolism, and rumen fermentation parameters data using SPSS (2019) statistical software. Duncan’s multiple comparison tests were utilized to investigate noteworthy variations among the group data. Disparities were evaluated statistically at (*p* ≤ 0.05).

## Results

### Growth performance and digestibility

The effects of dietary *Salicornia europaea* L. extract on final body weight (FBW), body weight gain (BWG), feed intake (FI), and feed gain ratio (FGR) are presented in Table 2. The initial body weights of (3.5-month-old sheep) did not significantly differ among the treatment groups (average, 32.5 ± 2.8 kg), demonstrating that the animals were divided into the experimental groups in a fully random manner.

**Table 2:** Effects of *Salicornia europaea* L. extract on the growth performance of sheep.

Items	Levels %				*p*-value
	0	0.2	0.4	0.6	
Initial body weight (kg)	33.78 ± 3.33	33.63 ± 3.31	33.68 ± 3.05	33.88 ± 3.39	0.998
Final body weight (kg)	47.38 ± 3.00	47.81 ± 3.10	48.34 ± 3.06	48.23 ± 3.91	0.888
Body weight change (kg)	13.61 ± 1.80	14.18 ± 2.00	14.66 ± 0.88	14.35 ± 1.18	0.398
Average daily gain (g)	302.34 ± 39.95	315.19 ± 44.38	325.83 ± 19.63	318.98 ± 26.19	0.398
Total FI (kg)	80.35 ± 6.95^a^	78.60 ± 5.98^a^	71.84 ± 6.12^b^	79.46 ± 9.34^a^	0.024
Average daily FI (kg)	1.79 ± 0.15^a^	1.75 ± 0.13^a^	1.60 ± 0.14^b^	1.76 ± 0.21^a^	0.024
Feed/gain ratio	6.02 ± 1.11^a^	5.65 ± 0.92^ab^	4.91 ± 0.47^b^	5.58 ± 0.82^ab^	0.025

^a,b^Means within the same row with different superscripts are significantly different (*P* ≤ 0.05).

The findings of the ANOVA revealed that there were no significant variations between the experimental groups’ FBW and BWG treatments, but there were substantial differences (*p* ≤ 0.05) between the experimental groups’ FI and FGR treatments. This suggests that applying different dosages of *Salicornia europaea* substantially enhanced the FI and FGR of sheep when compared with the control group. Furthermore, when compared to the other treatments, animals fed a diet that included 0.4% *Salicornia europaea* displayed better values of FI and FGR.

Table 3 shows the impact of nutritional supplements *Salicornia europaea* L. extract on DM, OM, CP, ADF, and NDF. Analysis of variance detected significant differences among treatments in the apparent digestibility of DM, OM, CP, ADF, and NDF. Sheep fed *Salicornia europaea* at 0.2, 0.4, and 0.6% diet levels had significantly higher levels than those in control.

**Table 3:** Effects of *Salicornia europaea* L. extract on apparent digestibility of nutrients in sheep feed.

Items	Levels %				*P*-value
	0	0.2	0.4	0.6	
Dry matter, %	55.85 ± 2.34^c^	59.34 ± 2.17^b^	62.50 ± 1.34^a^	63.25 ± 2.42^a^	0.001
Organic matter, %	59.87 ± 2.06^c^	62.86 ± 2.17^b^	66.10 ± 1.24^a^	67.01 ± 2.62^a^	0.001
Crude protein, %	70.08 ± 1.60^b^	72.14 ± 4.70^ab^	74.76 ± 2.85^a^	76.30 ± 3.39^a^	0.042
Neutral detergent fiber	38.43 ± 3.52^c^	43.96 ± 4.48^b^	50.94 ± 2.03^a^	55.03 ± 2.01^a^	0.001
Acid detergent fiber	19.61 ± 2.48^b^	22.31 ± 3.25^b^	32.48 ± 7.83^a^	29.23 ± 3.10^a^	0.002

^a,b,c^Means within the same row with different superscripts are significantly different (*p* ≤ 0.05).

### Rumen fermentation

Findings for *Salicornia europaea* L. extract used in meals were displayed in Table 4, along with its impact on pH, NH_3_-N, volatile fatty acids (VFA), butyrate, valerate, iso-valerate, and acetate/propionate.

**Table 4:** Effects of *Salicornia europaea* L. extract on rumen fermentation parameters of sheep.

Items	Levels %				*P*-value
	0	0.2	0.4	0.6	
pH	6.77 ± 0.43	6.99 ± 0.29	7.19 ± 0.32	6.99 ± 0.13	0.268
NH3-N, mg/dL	23.15 ± 2.59	22.48 ± 2.06	24.14 ± 3.15	23.66 ± 4.23	0.852
Total volatile fatty acids, mmol/L	99.71 ± 26.32	94.44 ± 16.47	85.60 ± 8.91	75.29 ± 15.47	0.190
Molar percentages of individual volatile fatty acids (%)					
Acetate	55.56 ± 6.12	53.92 ± 4.50	54.78 ± 2.26	54.84 ± 1.67	0.936
Propionate	30.84 ± 4.96	28.27 ± 4.89	30.93 ± 3.24	29.66 ± 3.85	0.741
Butyrate	10.36 ± 1.84	13.00 ± 2.60	10.39 ± 3.50	10.86 ± 2.39	0.372
Iso-butyrate	0.56 ± 0.15^b^	0.71 ± 0.27^b^	0.60 ± 0.16^b^	1.11 ± 0.49^a^	0.037
Valerate	1.90 ± 0.72	3.09 ± 1.69	2.29 ± 0.95	1.98 ± 0.44	0.300
Iso-valerate	0.78 ± 0.24	1.02 ± 0.45	1.01 ± 0.61	1.55 ± 0.70	0.167
Acetate/propionate	1.87 ± 0.58	1.98 ± 0.53	1.79 ± 0.19	1.87 ± 0.26	0.916

^a,b^Means within the same row with different superscripts are significantly different (*P* ≤ 0.05).

The results of the ANOVA showed that the ruminal pH and NH_3_-N concentration in fattening sheep had no significant variations in the experimental groups. The molar percentages of several of the individual volatile fatty acids, like propionate, acetate, butyrate, valerate, iso-valerate, and acetate/propionate, were also unaffected by the *Salicornia europaea* L. extract. The percentage of iso-butyrate in the 0.6% and control groups was substantially greater (*p* ≤ 0.05). As the amount of *Salicornia europaea* L. extract was added, the total volatile fatty acids in each group were gradually reduced.

### A synopsis of the sequencing information

Utilizing rumen digesta and fecal content samples, we performed 16S RNA gene sequencing to investigate the regulatory impact of *Salicornia europaea* L. extract on the sheep rumen microbiota. The rumen microbial alpha diversity indexes, such as Chao1, Goods coverage, observed species, Shannon, and Simpson, did not differ substantially, as Table 5 illustrates.

**Table 5:** Effects of *Salicornia europaea* L. extract on the α diversity of gastrointestinal bacteria communities in sheep.

Items	Levels %				*P*-value
	0	0.2	0.4	0.6	
Ruminal bacteria communities					
Chao1	3599.78 ± 571.89	3422.55 ± 701.99	3434.43 ± 559.47	3346.04 ± 287.43	0.903
Goods coverage	0.977 ± 0.004	0.982 ± 0.002	0.979 ± 0.007	0.981 ± 0.004	0.378
Observed species	3214.92 ± 342.83	3209.32 ± 367.08	3011.34 ± 360.16	2932.66 ± 266.97	0.466
Shannon	8.61 ± 0.62	8.94 ± 0.80	8.97 ± 0.51	8.65 ± 0.59	0.732
Simpson	0.977 ± 0.014	0.987 ± 0.005	0.988 ± 0.008	0.986 ± 0.008	0.305
Intestinal bacterial communities					
Chao1	3216.21 ± 292.94	3153.66 ± 352.77	3265.89 ± 294.45	3024.83 ± 433.90	0.722
Goods coverage	0.986 ± 0.003^ab^	0.99 ± 0.004^a^	0.983 ± 0.005^b^	0.988 ± 0.003^ab^	0.028
Observed species	2904.10 ± 280.63	2976.18 ± 314.57	3030.28 ± 167.74	3019.68 ± 209.10	0.851
Shannon	9.40 ± 0.65^ab^	9.37 ± 0.33^ab^	9.57 ± 0.31^a^	8.86 ± 0.55^b^	0.148
Simpson	0.988 ± 0.017	0.993 ± 0.004	0.994 ± 0.002	0.988 ± 0.007	0.588

^a,b^Means within the same row with different superscripts are significantly different (*P* ≤ 0.05).

The intestinal bacterial α-diversity indices, such as the Chao1, Goods coverage, and observed species (*p* ≤ 0.05), may be impacted by the *Salicornia europaea* L. extract. Compared to the 0% groups, the Chao1, and observed species were reduced (*p* ≤ 0.05) in the 0.6% group. Also, in the control groups, the Goods coverage increased (*p* ≤ 0.05) in the 0.6% group.

### Analysis of discrepancies

**Figure 1:**
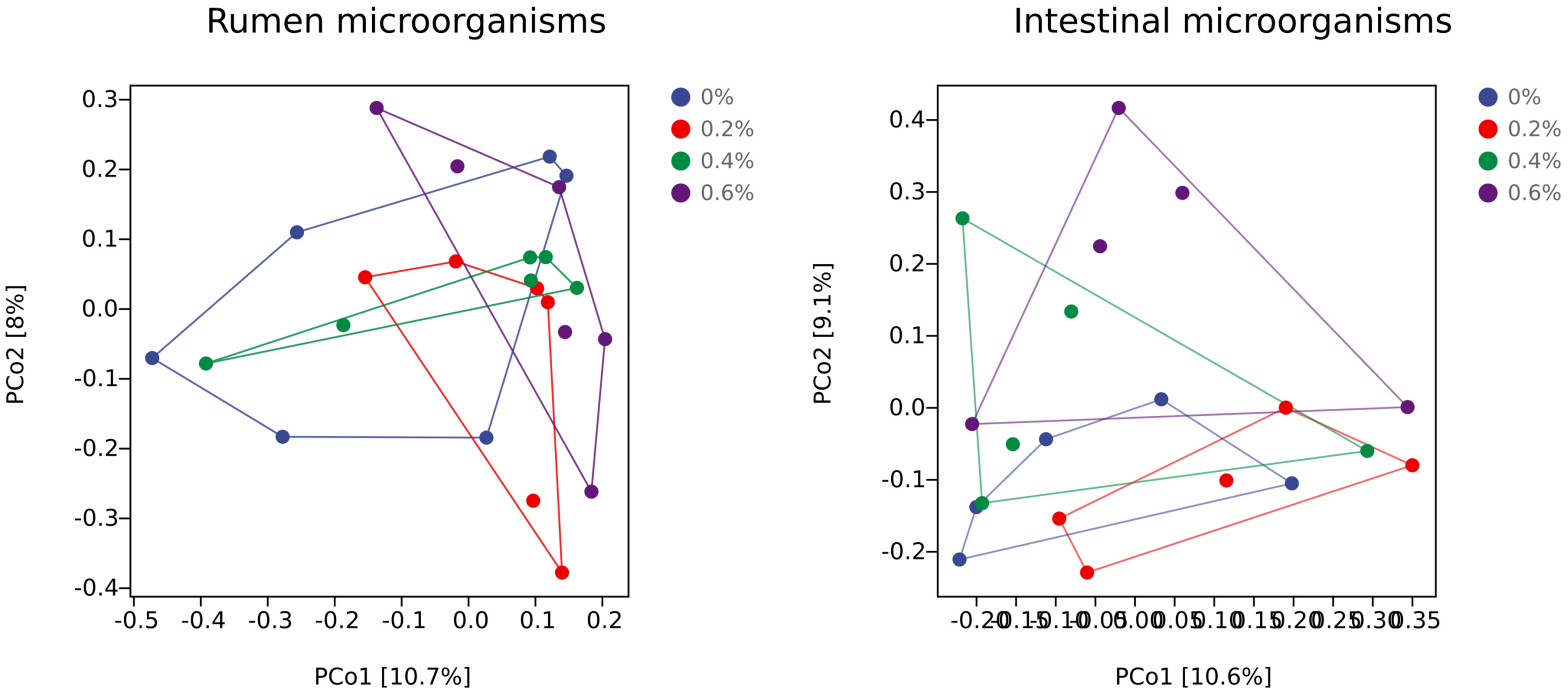
Changes in prokaryotic community composition visualized as a principal coordinate analysis (PCoA) using Bray–Curtis dissimilarity metrics. The percentage of variation explained is indicated on the respective axes and ellipses to illustrate the 95% confidence intervals.

Principal coordinate analysis (PCoA) can show disparities between individuals or groups. The first principal component, or PCo1, represented 10.7% of the variance of the separated samples. The second principal component, or PCo2, represents the separated sample’s 8% variance. Figure 1A shows that the 0.6% group’s aggregation degree is higher than the control groups, suggesting that including 0.6% *Salicornia europaea* L. extract in the diet can increase the group’s microbial flora composition similarity. The 0.6% group and the control group were greatly separated, suggesting that the microbial flora in the rumen fluid had a very different structure.

The PCoA of Figure 1B shows how the intestinal microflora varies amongst individuals or groups. The first principal component, or PCo1, accounted for 10.6% of the variation in the divided samples. With PCo2 accounting for 9.1% of the variance of the separated samples, it is the second principal component. The 0.4% group’s aggregation degree was higher than that of the control group, suggesting that including 0.4% *Salicornia europaea* L. extract in the diet may have increased the group’s flora composition similarity. The 0.6% group and the 0.2% control group were separated by a considerable amount, suggesting that the intestinal microflora’s structure was significantly different.

**Figure 2:**
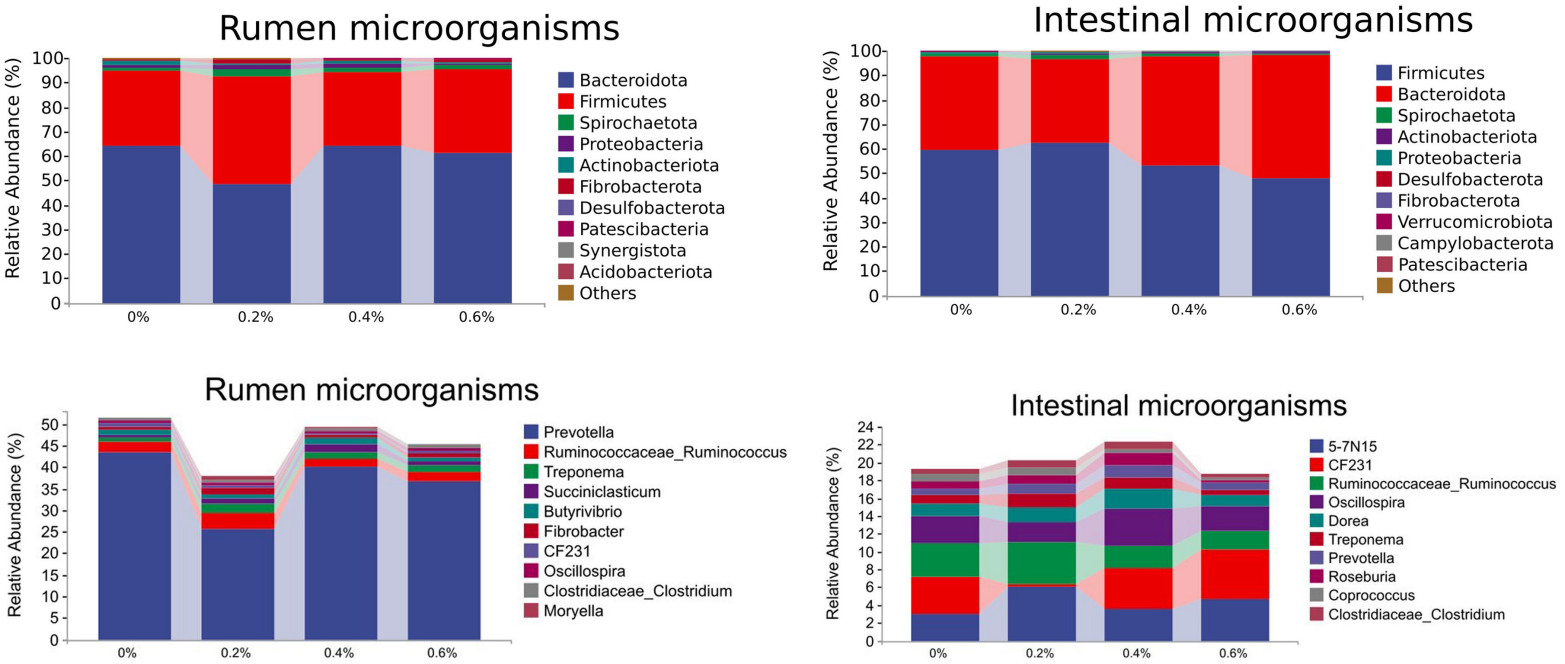
Gastrointestinal microorganisms of relative abundance at the phylum and genus level.

Figure 2A depicts the proportional distribution of rumen flora in the various treatment groups. Of these, 95.79% or more were accounted for by Bacteroidetes (58.86% ~ 67.34%), Firmicutes (27.86% ~ 49.45%), Spirochaetes (0.69% ~ 2.54%), and Proteobacteria (0.40% ~ 2.53%), which was the predominant phylum in sheep rumen.

Figure 2B illustrates the corresponding proportions of rumen bacteria in the various treatment groups. *Prevotella, Ruminococcus, Treponema, Succihiclasticum,* and *Butyrivibrio* were the most common genera in sheep rumen, accounting for 39.91% or more of the total relative abundance of the bacterial phylum. A difference of statistical significance was observed at *p* < 0.05 in the Treponema levels between the 0.2% and control groups.

Figure 2C displays the relative abundance of intestinal microbial phylum levels in each treatment group. Of them, the following are Spirochaetes (0.56% ~ 1.65%), Bacteroidetes (31.92% ~ 48.20%), and Firmicutes (49.81% ~ 64.54%). The two phyla that dominated sheep rumen, Firmicutes, and Bacteroidetes, accounted for 96.46% and more of the phylum’s total relative abundance. Furthermore, in the 0, 0.2, 0.4, and 0.6% groups, the percentages of Firmicutes and Bacteroidetes were 96.46, 98.01, 96.46, and 96.77%, respectively. The amount of *Salicornia europaea* L. extract added increased along with the number of Firmicutes and Bacteroidetes in the sheep’s posterior digestive tract.

Figure 2D displays the proportional distribution of the intestinal microbial flora in the various treatment groups. The predominant genera in the sheep intestine, 5-7 N15, CF231, *Ruminococcus,* and *Oscillospira*, accounted for 12.16% or more of the total relative abundance of bacterial phyla.

### Phylogenetic investigation of communities

**Figure 3:**
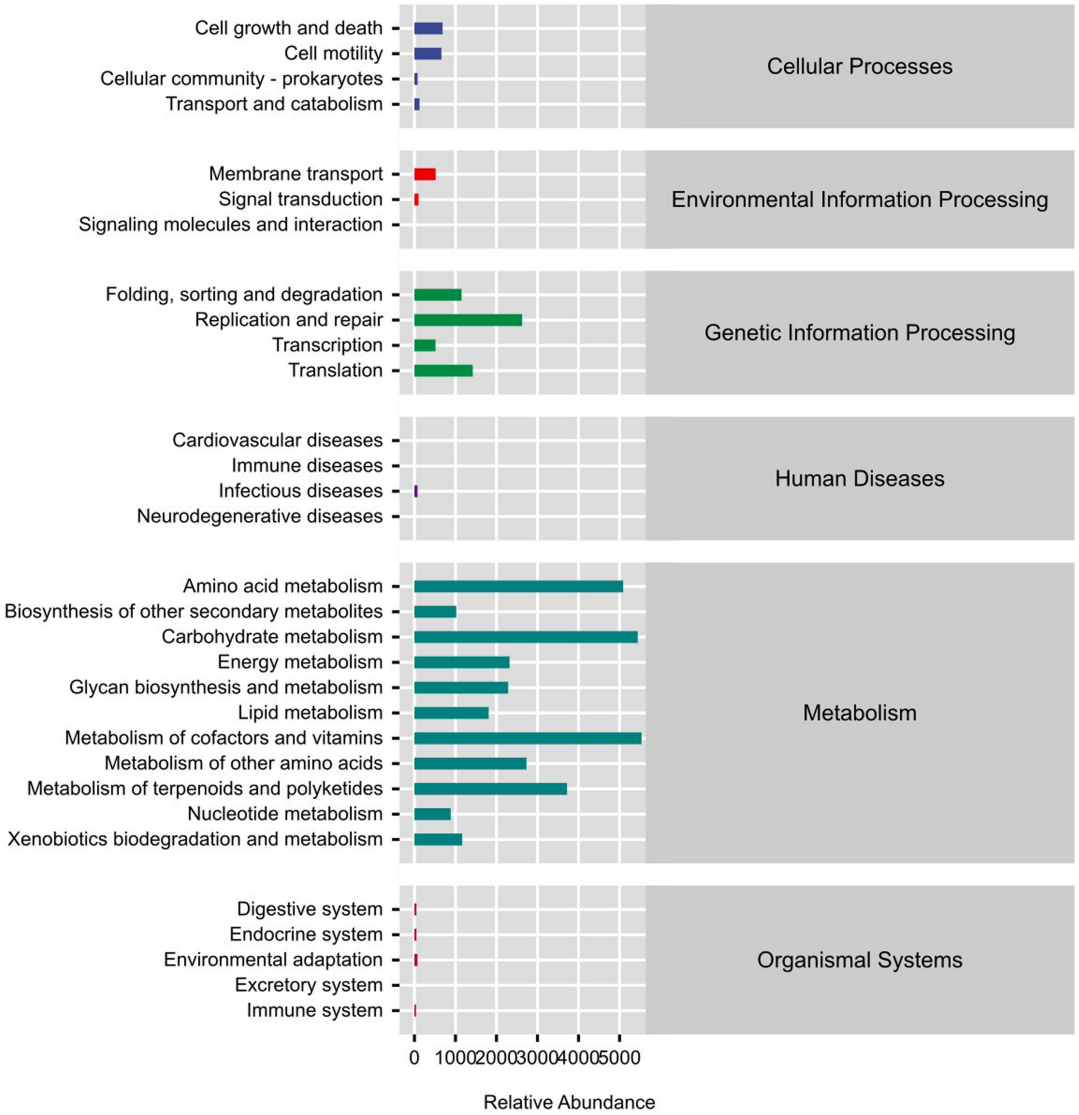
KEGG metabolic pathway prediction frame line diagram.

In the study, Phylogenetic Investigation of Communities by Reconstruction of Unobserved States (PICRUSt2) software was used to predict the KEGG (Kyoto Encyclopedia of Genes and Genomes) pathway that may exist in the sequencing results of sheep rumen fluid samples. As can be seen in Figure 3, the microbial functional gene KEGG enrichment pathway framework diagram is primarily concerned with the metabolism of amino acids, carbohydrates, cofactors, vitamins, glycans, nucleotides, lipids, and other substances. Metagenome Sequencing analysis and comparison, based on the outcomes of PICRUSt2 functional secondary classification, revealed the absence of any significant up-regulated or down-regulated metabolic pathways. The experimental group exhibited higher levels of terpenoids and polyketide metabolism, as well as biodegradation and xenobiotic metabolism (Figure 3), compared to the control group. This difference could potentially be attributed to the coexistence of multiple microbial functions.

**Figure 4:**
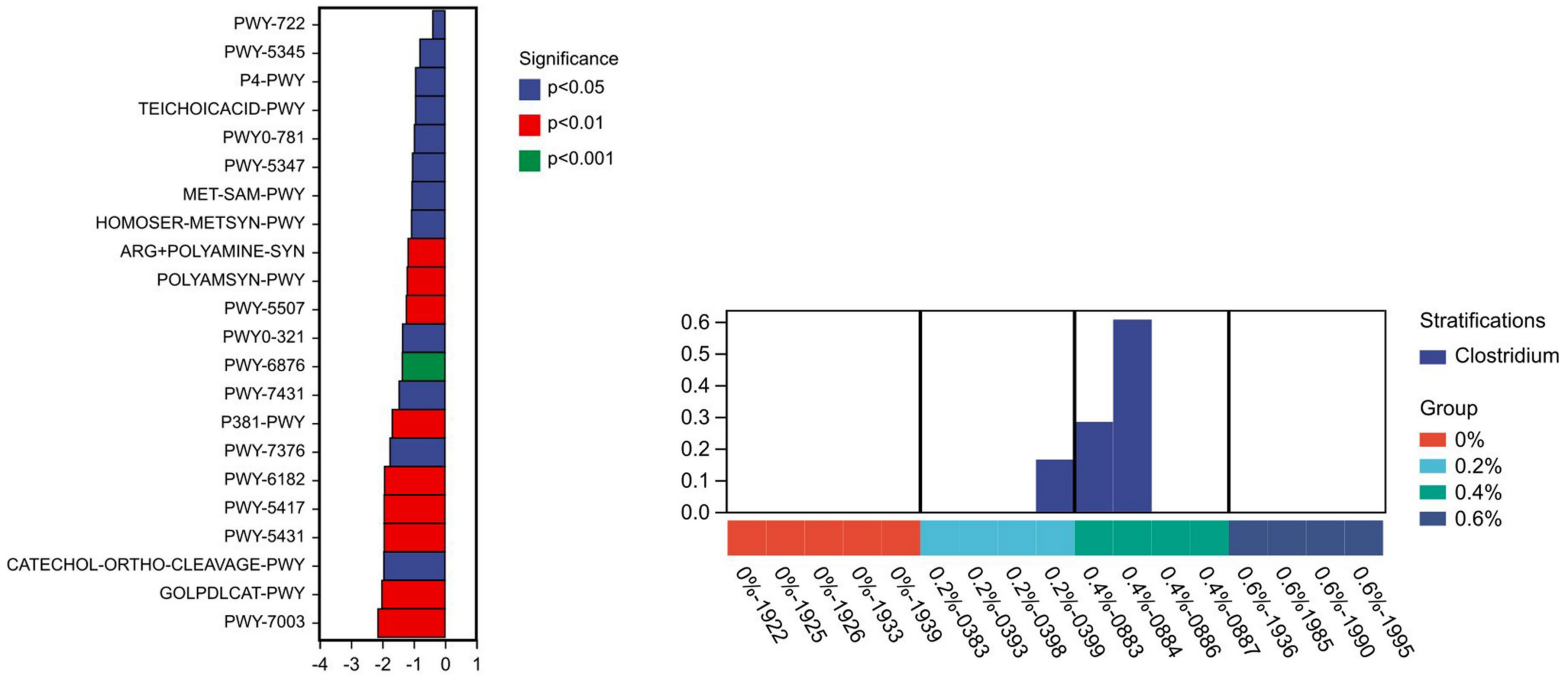
Analysis of intergroup differences in metabolic pathways.

The KEGG enrichment pathway block diagram of microbial functional genes, as shown in Figure 3, is primarily associated with amino acids metabolism, carbohydrates, cofactors, and vitamins, as well as the biosynthesis and metabolism of glycans, nucleotide metabolism, lipid metabolism, the immune system, and the digestive system. The cofactor abundance, vitamin metabolism, glycan biosynthesis, nucleotide metabolism, and digestive system metabolism were higher in the 0.6% group than in the control group. The metagenome Seq method was utilized to analyze and compare the outcomes based on the PICRUSt2 functional secondary classification results. The metabolic pathway was found to be primarily contributed by Clostridium, with the PWY-6876 (isopropanol biosynthesis) pathway being the most significantly down-regulated (*p* ≤ 0.001) (Figure 4). It might be because *Salicornia europaea* extract affects Clostridium in a particular way.

## Discussion

Our results showed no significant differences between experimental groups in FBW and BWG, but significant differences in FI and FGR between treatments. Different *Salicornia europaea* dosages enhanced sheep’s FI and FGR, with a 0.4% diet showing better results. According to Sadeghi et al. (2020), substituting *Halocnemum* and *Suaeda* for wheat straw and alfalfa hay in high-concentrate diets can maintain fattening performance and decrease the fat deposited on lamb carcasses. Also, Holguin Peña et al. (2020) believe *Salicornia* hay-based forage can be utilized as a forage component but is not necessary for the Creole goat’s diet because it promotes the growth and milk production of the goat offspring. Likewise, Marwan et al. (2018), producing halophyte feed (kochia) with saline water may be a way to make up for the lack of feed supplies, reduce feed costs, and boost the profitability of raising rabbits.

According to Jiao et al. (2019), using *Salicornia herbacea* (5 cc/L, 10 cc/L) improved the broiler’s BWG and FCR and changed the carcass quality by lowering the level of abdominal fat. This suggests that broiler supplementation with 5 cc/L of *Salicornia herbacea* might be adequate to attain the best possible response in terms of meat quality and growth efficiency. Al-Batshan et al. (2008) noted that *Salicornia bigelovii* was useful in lowering FI, and consequently BWG and FBW in broilers. According to research by Mohammadi et al. (2015), laying hens given 1 cc and 5 cc of *Salicornia herbacea* extracts per liter of drinking water produced more eggs and broke them less frequently, with no apparent distinctions between the two treatments. Conversely, Sarker et al. (2010) found that supplementing broiler chicks’ feed with a *Salicornia herbacea* diet (0.5, 1.0%) had no substantial impact on their BWG, FCR, or FI. According to Kim et al. (2006), giving rats regular diets supplemented with 2% enzyme-treated *Salicornia herbacea* extracts did not significantly alter the rats’ BWG, FI, or food effectiveness ratios. Since NaCl is the primary component of salicornia, reducing FI is necessary because high sodium levels in the diet increase water intake and the flow of undigested digest in the rumen, which enhances protein digestion, lowers fiber fermentation, and lowers FI.

Our results reveal significant differences in digestibility of DM, OM, CP, ADF, and NDF among treatments, with sheep fed *Salicornia europaea* at varying levels showing higher levels. According to Taghipour et al. (2021), adding *Salicornia bigelovii* forage up to 30% of it instead of alfalfa hay to a sheep’s maintenance diet enhanced the blood’s total antioxidant activity and reduced *in vitro* ruminal production of methane without hurting the nutrient digestion of the animal *in vivo*. Additionally, the animals’ salt intake was lower (60.4 g/kg of diet DM) than the permissible level (70 to 100 g/kg of DM), according to the NRC (2005), which may have hurt feed intake. However, as the amount of salt (ash) consumed increased, the addition of *Salicornia bigelovii* forage substantially reduced daily OM intake (OMI) (Kraidees et al., 1998). Consequently, if the results are to be utilized in comparing halophytes (like Salicornia) to other forages, an adjustment for the amount of ash in *Salicornia bigelovii* forage must be made after calculating the insoluble ash to determine nutrient digestibility (Masters et al., 2001). According to El Shaer (2010), the median CP of the *Salicornia* species is 130 g/kg DM. Furthermore, Bañuelos et al. (2018) stated *Salicornia* has a low lignin content of 19.6 g/kg on a DM basis. Seven distinct halophyte plants were examined by Akinshina (2014), who found that the ash content ranged from 140 to 490 g/kg DM. Masters et al. (2007) indicated that increased osmotic pressure in the digestive tract due to greater ash (including salt) in *Salicornia bigelovii* forage meals may reduce the digestibility of nutrients and digestible organic matter in the diet by shortening the ruminal nutrients retention time and the activity of bacteria. However, when fed a mixed diet with saltwort at a 20% inclusion level on a dry matter basis, no negative impact on feed intake was observed in native Japanese goats (Shimizu et al., 2001). In contrast, Mahmoud et al. (2016) discovered that dietary crude protein concentrations above 14.5% of dry *salicornia* biomass did not enhance performance or carcass quality fatness in young Majaheem male camels, nor was 14.5% crude protein enough for the animals’ nutrition efficiency and adiposity. For small ruminant production systems, the *Salicornia europaea* L. extract (0.4, 0.6% DM) treatment showed improved CP digestibility and is natural, sustainable, and safe.

According to our data, the ruminal pH and NH_3_-N levels were not substantially different between the trial groups, and the *Salicornia europaea* L. extract did not affect the molar ratios of volatile fatty acids other than iso-butyrate. A possible explanation for this decreasing NH_3_-N level as *Salicornia europaea* levels rise is a possible rise in microbial nitrogen synthesis or the increased uptake of NH_3_-N by the microbial biomass. Our results agree with Taghipour et al. (2021), who reported that the addition of *Salicornia bigelovii* forage did not affect the ruminal pH, levels of volatile fatty acids, or the ratio of acetate to propionate *in vivo*. Also, Degen and Squires (2015) stated that dietary inclusion reduces total protozoa and Entodiniinae numbers, indicating that *Salicornia bigelovii* forage diets and salt load may impact rumen microbiota. According to Belanche et al. (2016), the abundance of anti-protozoal compounds (tannins) may have contributed to the decrease in ruminal protozoal groups in *Salicornia bigelovii* forage-fed sheep. According to Abouheif et al. (2000), Salicornia biomass in the diets of Najdi rams changed patterns of fermentation and digestion, possibly increasing the amount of undegraded nutrients. On the other hand, the availability of freshwater had no detrimental effects on nitrogen retention or nutritional value. Also, Nasrabadi et al. (2022) found that replacing alfalfa hay with Salicornia forages in shallow male sheep diets increased antioxidant capacity, without affecting nutrient digestibility or methane production.

Our findings showed that *Salicornia europaea* L. extract could influence intestinal bacterial α-diversity indices by enhancing Goods coverage in the control group and decreasing Chao1 and Observed species in the 0.6% group. According to Cui et al. (2019), the sheep-fed oats had higher Goods-coverage, Shannon-Wiener index, Simpson index, ACE, and Chao 1 index than the sheep-fed native pasture. These variations were all substantially greater (*p* < 0.05). There was no statistically significant variance in the goods coverage of OTUs between the two groups (*p* > 0.05). Additionally, it is widely recognized that the rumen microbiota’s composition varies depending on the stage of growth. The rumen microbiota of yak from birth to 12 years of age demonstrated changes associated with age and maturation (Han et al., 2015). However, research by Wang et al. (2018) on goats revealed that age and different nutrients had a major impact on the rumen microbial diversity.

The taxa affected by dietary changes exhibit a significant change at the genus level. According to our findings, the most prevalent genera in sheep rumen were variations of the genus *Prevotella, Ruminococcus, Treponema, Succihiclasticum,* and *Butyrivibrio*. There was a statistically significant difference between the 0.2% and control groups (*p* < 0.05) in the Treponema levels. Our findings, however, are not consistent with earlier studies (Cui et al., 2019; Wang et al., 2019) that indicated *Prevotella* to be the most abundant genus. Also, Domínguez et al. (2022) found that the genus *Ruminococcaceae,* which is associated with the degradation of cellulose, had the highest dominance across all samples. *Christensenellaceae, Rikenellaceae,* and *Prevotellaceae* were the following most prevalent genera. In contrast to adult goats fed Salicornia, Campylobacter surprisingly predominates.

After examining the rumen bacterial community of Tibetan sheep in the Qinghai Tibetan Plateau, Cui et al. (2019) found that the two most prevalent phyla, accounting for 53.72 and 43.64% of the overall bacterial abundance, were *Bacteroidetes* and *Firmicutes*. It is in line with numerous earlier herbivore studies (Hook et al., 2011). The metabolism of fiber, protein, and carbohydrates is intimately linked to the activities of *Firmicutes* and *Bacteroidetes* (Huo et al., 2014). The primary components of crop straw are cellulose, hemicellulose, and lignin. In contrast, the Firmicutes contained many bacteria that break down fiber, including *Butyrivibrio, Ruminococcus, Pseudobutyrivibrio, Oscillibacter,* and *Eubacterium*. This helps to explain why the Firmicutes completely dominate the rumen microflora of ruminants (Hook et al., 2011).

## Conclusion

The study investigated suggests adding the effect of *Salicornia europaea* L. extract to the bacterial population in the sheep’s gastrointestinal tract, rumen fermentation parameters, growth performance, and nutrient digestibility. When provided at 0.4% DM, we discovered that dietary *Salicornia europaea* L. extract reduced feed to feed-to-gain ratio but did not affect growth performance. The 0.4 and 0.6% treatments demonstrated increased CP digestibility. *Salicornia europaea* L. extract is safe, natural, and sustainable for small ruminant production systems because it increases acetic acid in rumen fluid, reduces propionic acid, and decreases total volatile fatty acids.

## Data availability statement

The datasets presented in this study can be found in the NCBI repository, accession numbers PRJNA1094564 and PRJNA1094575.

## Ethics statement

The protocols and methods used in the experiments were authorized by the Xinjiang Animal Welfare and Ethics Committee Academy of Animal Sciences, China (Approval No., 2021–276, 15 May 2021). The studies were conducted in accordance with the local legislation and institutional requirements. Written informed consent was obtained from the owners for the participation of their animals in this study.

## Author contributions

MK: Conceptualization, Data curation, Formal analysis, Funding acquisition, Investigation, Methodology, Project administration, Resources, Software, Supervision, Validation, Visualization, Writing – original draft, Writing – review & editing. WL: Conceptualization, Data curation, Formal analysis, Funding acquisition, Investigation, Methodology, Project administration, Resources, Software, Supervision, Validation, Visualization, Writing – original draft, Writing – review & editing. TS: Conceptualization, Data curation, Methodology, Resources, Writing – original draft. LJ: Data curation, Formal analysis, Investigation, Methodology, Resources, Writing – original draft. QR: Formal analysis, Investigation, Methodology, Resources, Writing – original draft. LY: Conceptualization, Funding acquisition, Project administration, Resources, Supervision, Visualization, Writing – review & editing. WW: Funding acquisition, Methodology, Software, Supervision, Validation, Visualization, Writing – review & editing. CX: Investigation, Methodology, Project administration, Resources, Writing – original draft. YC: Investigation, Validation, Visualization, Writing – review & editing, Funding acquisition, Supervision.
